# Detection of Relative Afferent Pupillary Defect and Its Correlation with Structural and Functional Asymmetry in Patients with Glaucoma Using Hitomiru, a Novel Hand-Held Pupillometer

**DOI:** 10.3390/jcm12123936

**Published:** 2023-06-08

**Authors:** Makoto Nakamura, Mari Sakamoto, Kaori Ueda, Mina Okuda, Fumio Takano, Yuko Yamada-Nakanishi

**Affiliations:** Division of Ophthalmology, Department of Surgery, Graduate School of Medicine, Kobe University, 7-5-2 Kusunoki-cho, Kobe 650-0017, Japan

**Keywords:** relative afferent pupillary defect, optical coherence tomography angiography, hand-held pupilometer, asymmetrical glaucomatous damage

## Abstract

Patients with asymmetric glaucomatous optic neuropathy (GON) present a relative afferent pupillary defect (RAPD) in the eye with more advanced damage. Although useful, pupillometric RAPD quantification is not widely used as it is not portable. Whether asymmetry of the peripapillary capillary perfusion density (CPD) detected using optical coherence tomography angiography correlates with the severity of RAPD remains unknown. This study assessed RAPD in 81 patients with GON using Hitomiru, a novel hand-held infrared binocular pupillometer. The correlation and ability to detect clinical RAPD based on the swinging flash light test of two independent RAPD parameters (the maximum pupil constriction ratio and the constriction maintenance capacity ratio) were assessed. The coefficient of determination (R^2^) was calculated between each of the two RAPD parameters and asymmetry of the circumpapillary retinal nerve fiber layer thickness (cpRNFLT), ganglion cell layer/inner plexiform layer thickness (GCL/IPLT), and CPD. The two RAPD parameters showed a correlation coefficient of 0.86 and areas under the receiver operating characteristic (ROC) curve of 0.85–0.88, with R^2^ being 0.63–0.67 for the visual field, 0.35–0.45 for cpRNFLT, 0.45–0.49 for GCL/IPLT, and 0.53–0.59 for CPD asymmetry. Hitomiru has high discriminatory performance in detecting RAPD in patients with asymmetric GON. CPD asymmetry may better correlate with RAPD than cpRNFLT and GCL/IPLT asymmetry.

## 1. Introduction

A relative afferent pupillary defect (RAPD) represents the left–right asymmetric impairment of the anterior visual input pathway from the retina to the lateral geniculate nucleus [1,2]. RAPD is an important ophthalmologic biomarker because of its objectiveness, unlike subjective functional tests such as the visual acuity and visual field, which are dependent on the patient response. Glaucoma is the degenerative loss of retinal ganglion cells and optic nerves and often develops and progresses asymmetrically [3,4]. The incidence of RAPD in patients with glaucoma varies (9–82%) and depends on the method of detection, definition of RAPD, and enrollment criteria of subjects [5].

Clinically, RAPD is evaluated by the swinging flashlight test (SFLT) using neutral density filters [2] and reportedly correlates with asymmetrical visual field loss and retinal structural changes [6,7]. Meanwhile, electronic pupillometry with infrared monitoring is used for more precise pupillary response recording [8,9]. The quantification of RAPD in glaucoma by electronic pupillometry has also been attempted [10,11,12,13,14]. However, RAPDx^®^ (Konan Medical, Nishinomiya, Japan) [11,12,13,14] is a stationary device that is not portable. The proprietary RAPD evaluation index built into RAPDx^®^, termed the RAPD score, is calculated based on the ratio of the pupil diameter at maximum constriction to the baseline pupil diameter during brief stimulation and is not identical to RAPD as determined by SFLT. Several portable electronic pupillometers have been developed [15,16,17], but most are used for monocular observation and are not suitable for RAPD evaluation.

The novel Hitomiru electronic pupillometer (Uratani Lab, Osaka, Japan), which allows for the simultaneous recording of the binocular pupillary response, is portable, convenient, and useful in emergency medicine [18]. Additionally, Hitomiru may potentially quantify RAPD because the user is free to set the stimulation and observation programs.

The degree of RAPD has been reported to correlate with the left–right ratio of the circumpapillary retinal nerve fiber layer thickness (cpRNFLT) [6,7,11,14], retinal ganglion cell layer/inner plexiform retinal layer thickness (GCL/IPLT) at the macula [7,14], or related parameters [13] as measured by optical coherence tomography (OCT). Recent developments in OCT angiography (OCTA) have gained attention due to their use in determining the relationship between peripapillary and macular vascular changes (capillary density and peripapillary choroidal capillary structure) and glaucomatous optic neuropathy (GON) [19]. However, no reports have evaluated the correlation between parameters obtained by OCTA and RAPD.

This study aimed to determine whether Hitomiru can detect RAPD in patients with asymmetric GON. In addition to the RAPD score used in RAPDx^®^, we developed a new evaluation index that mimics the detection of RAPD by SFLT and examined the ability of both indices to detect RAPD identified by SFLT. Additionally, we examined the correlation between RAPD indices in Hitomiru and asymmetry of subjective visual dysfunction with the OCT and OCTA parameters.

## 2. Materials and Methods

### 2.1. Subjects

This cross-sectional study was approved by the Medical Ethics Committee of Kobe University Graduate School of Medicine (Permission No. B220128) and conducted in compliance with the Declaration of Helsinki. Since this was a non-invasive observational study, it was ethically acceptable to obtain verbal informed consent from each patient after explaining the nature of the study.

Subjects included 81 non-consecutive patients who underwent both SFLT and pupillary function testing using Hitomiru between 1 June 2021 and 31 May 2022. All subjects underwent full clinical ophthalmological evaluation, including Landolt visual acuity testing, Goldman applanation tonometry, slit-lamp stereo biomicroscopy, indirect ophthalmoscopy, Humphrey visual field (HVF) testing, OCT, and OCTA. Best corrected visual acuity was converted to the logarithm of minimum angle of resolution (logMAR) for statistical analysis.

The inclusion criteria encompassed patients ≥18 years old with primary open-angle glaucoma (including preperimetric glaucoma) or ocular hypertension that had good-quality data from HVF and OCT and OCTA performed within 3 months of recording the pupillary response. Patients with either one of the following conditions were excluded: (1) a good pupil response could not be obtained (i.e., due to excessive blinking), (2) systemic autonomic nervous system diseases affecting the efferent pathway of the light reflex such as diabetes existed, (3) pathological anisocoria such as oculomotor nerve palsy, (4) intraocular diseases affecting the accurate evaluation of the light reflex (i.e., corneal opacity, mature cataract, uveitis, etc.) existed, (5) primary angle-closure glaucoma and secondary glaucoma including exfoliation glaucoma, and (6) previous intraocular surgery except for uncomplicated cataract surgery that had been performed at least 6 months before this study.

Glaucoma was diagnosed based on structural abnormalities of the optic disk and associated visual field defects as detected by the HVF test (Carl Zeiss Meditec, Jena, Germany) using the 24-2 Swedish Interactive Thresholding Algorithm standard [20]. Results were considered reliable when the fixation loss was <20% and the error rate of false positives and false negatives was <25%. The criteria proposed by Anderson and Patella were used to determine glaucomatous visual field impairment [21]. Patients with ocular hypertension were defined as those with an intraocular pressure of ≥21 mmHg but without glaucomatous optic disk changes and the aforementioned visual field abnormalities. Patients with preperimetric glaucoma were defined as those with glaucomatous optic disk changes but without the aforementioned visual field abnormalities. The mean deviation (MD) and visual field index (VFI) were used for analysis.

SFLT was performed by an examiner (Y.Y.-N.) with sufficient experience [6]. The examiner was blinded to the results of the Hitomiru pupillometry and visual field and OCT and OCTA examinations. Patients were instructed to fixate on a distant target in a mesopic room (the luminance = 10 lux) for 60 s in a sitting position. Using an indirect ophthalmoscope, each eye was then illuminated for 2 s from a light source viewing angle of 30°. Four to six alternating light stimuli were given. RAPD was present when any of the following findings were present: an initial slight momentary constriction of the pupil followed by mydriasis, an initial lack of pupillary movement followed by mydriasis, or mydriasis occurring immediately after light exposure [2,22].

### 2.2. Measurement Principle of the Hitomiru Electronic Pupillometer

**Figure 1:**
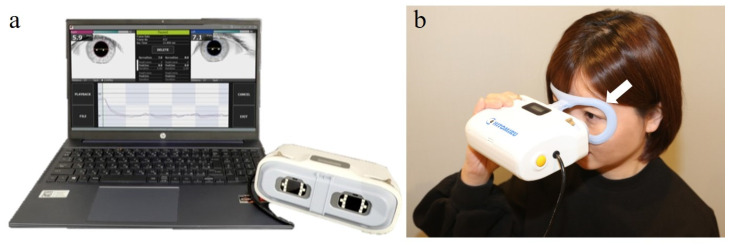
Appearance and recording images of the Hitomiru^®^ electronic pupillometer. (**a**) Hitomiru device appearance. (**b**) Examiner positioning during recording. White arrow: a plastic eyepiece frame attached to support the forehead so that the proper distance of 3 cm between the eye and the instrument can be maintained.

Hitomiru is a portable electronic pupillometer that allows for the simultaneous recording of bilateral light reflexes (Figure 1) [18]. The device weighs 477 g and is run by interfacing with the operating software installed on a personal computer. The optical unit is controlled by a circuit board that produces a real-time image of the pupil displayed in a 640 × 480 pixel array. Hitomiru projects white visible light onto the pupil of one eye to induce constriction and measures and monitors the shape and diameter of both pupils under infrared light using a short-range infrared detector with a horizontal angle of view of 90.2°. The detector is also used to measure the distance between the camera and the pupil and to correct for the measured diameter of each pupil.

A pair of near-infrared and white visible light emitting diodes (LEDs) are positioned at 23° and 21° from the center of the optical axis of the camera on the left and right sides, respectively, for a total of four LEDs. By attaching a special plastic pad to the device and fitting the eyebrow and cheekbone area to the pad, the distance between the camera and the pupil plane is fixed at approximately 3 cm. On the pupil plane, the irradiation area has a diameter of 36.2 mm for infrared light and 34 mm for white light. For pupillary response recording, a non-Maxwellian stimulus was positioned so that the entire pupil was always within the irradiated light beam, to prioritize portability and simulate clinical SFLT. For the same reason, no occluder was placed between the two cameras. Additionally, because Hitomiru is not a system that projects stimuli on a screen, a fixed target is not presented, so the patient was instructed to assume that they were looking into the distance and to keep their eyes still.

The peak wavelength and illuminance of the infrared and white LEDs were 860 nm and 0.36 lux, and 478 nm and 716.84 lux, respectively. The tests were conducted in a darkroom environment at illuminance of less than 0.5 lux, with background light of 780 nm and illuminance of 0.36 lux.

Twenty frames of pupil images were captured per second. After binarizing the images, the software recognized the size of the circle matching the pupil margin, and the horizontal diameter of the pupil was recorded and displayed as the number of pixels.

The raw data of the pupil diameter change (pixels) over time were exported to a computer algorithm for analysis. A curve representing the diameter relative to the baseline diameter over time was plotted. Since pixel values are directly related to the pupil diameter in millimeters (22.8 pixels = 1 mm), the pupillary diameter was calculated using pixel values. In some cases, the test had to be repeated due to blink artifacts. Responses contaminated by blinking or recording artifacts were excluded before data analysis.

### 2.3. RAPD Measurement and Analysis Method with Hitomiru

**Figure 2:**
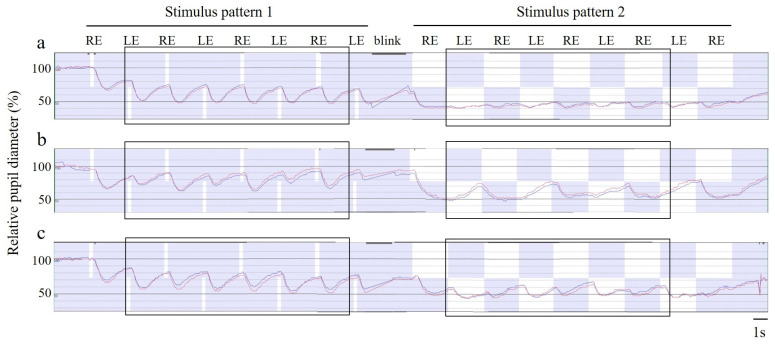
Representative examples of pupil response recording. Changes over time in two consecutive stimulus patterns are depicted for the relative pupil diameter, which was defined as the percentage of each eye’s horizontal pupil diameter when the average pupil diameter during the adaptation time after the start of recording was set to 100%. (**a**) A case with no relative afferent pupillary defect (RAPD) on the swinging flashlight test (SFLT) (see Supplemental Video S1). (**b**) A case with RAPD in the left eye (see Supplementary Materials Video S2). (**c**) A case with RAPD in the right eye (see Supplementary Materials Video S3). RE, right eye stimulation; LE, left eye stimulation. Red line, right eye reaction. Blue line, left eye reaction. The 1-s indication and bar in the lower right corner represent a 1-s time scale. RAPD scores 1 and 2 were calculated based on the responses from three stimulus pairs for each of the stimulus patterns enclosed in the black squares. (**a**) In stimulus pattern 1, the rate of pupil constriction change in both eyes is equivalent, and in stimulus pattern 2, both eyes show slight pupil constriction after the onset of stimulation and slight mydriasis at approximately the same level during the 2-s sustained stimulation period. (**b**) In both stimulus patterns 1 and 2, the pupil constriction responses of both eyes during left eye stimulation are smaller than those of right eye stimulation. (**c**) Meanwhile, it is difficult to capture the difference between the left and right pupil responses when observing only the amount of constriction of short-term stimuli such as stimulus pattern 1, but it is easier to grasp the difference between the left and right pupil responses when paying attention to the state of constriction maintenance during long-term stimuli, such as stimulus pattern 2.

Two sets of stimulus patterns were presented as a series of sequences. In both patterns, the right eye was stimulated first, then the left eye, and then the right eye again, in alternating order. After a 2-s no-stimulus period, the first pattern was presented, which consisted of a 200-ms stimulus presentation period and a 1.9-s no-stimulus observation period as a duty cycle; this was repeated four times for each eye, for a total of eight cycles. A 1-s interval was then followed by the presentation of a second pattern. In this pattern, after the 2-s stimulation of one eye, the opposite eye was alternately stimulated for 2 s without pause. After four rounds of this alternating stimulation, the recording was terminated with a preliminary 2-s right eye stimulation followed by a 2-s no-stimulus observation period (Figure 2, Supplementary Materials Videos S1–S3). The first stimulus pattern mimics RAPDx^®^ [11,12,13,14], while the second mimics clinical SFLT. The total recording time for the sequence was set to 39.8 s. Patients were instructed to blink between the last left eye stimulation of the first stimulus pattern and the start of the second stimulus pattern.

The pupillometer’s proprietary analysis software calculated the following parameters for each of the three pairs of responses for each stimulus pattern and then averaged them.

RAPD in the first stimulus pattern is an index of the direction and magnitude of asymmetry of the maximum contraction percentage, mimicking the RAPD score of RAPDx^®^ [11,12,13,14]. Referred to here as RAPD score 1, it is calculated using the following formula:

RAPD score 1 = 10 × log_10_ (od/os)

**Figure 3:**
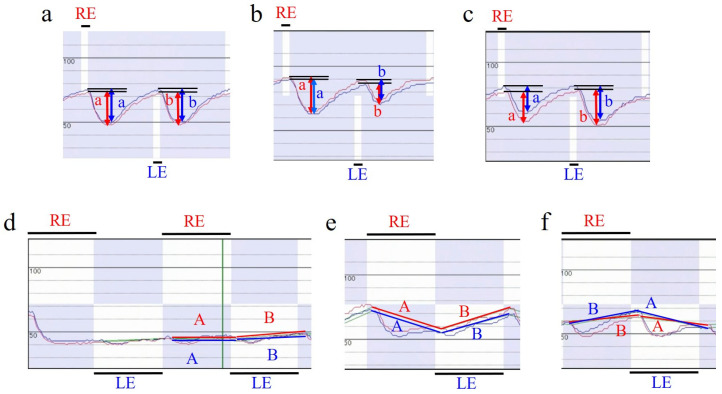
Calculation of RAPD scores 1 and 2. Red waves represent pupillary movement in the right eyes, whereas blue ones do in the left eyes. (**a**–**c**). The rate of change in pupil diameter utilized to obtain RAPD score 1 is shown for one pair of pupil responses in stimulus pattern 1 in cases a–c, respectively, in Figure 2. RE, 0.2-s stimulation of the right eye; LE, 0.2-s stimulation of the left eye. Colored a, the difference between the relative pupil diameter at the start of constriction and at maximum constriction during right eye stimulation. Colored b, the difference between the relative pupil diameter at the start of constriction and at maximum constriction during left eye stimulation. Red double arrow, pupil change in right eye; blue double arrow, pupil change in left eye. RAPD score 1 was calculated based on the formula 10 × log_10_[{(a in red + a in blue)/2} ÷ {(b in red + b in blue)/2}. (**d**–**f**). The rate of change in pupil diameter utilized to obtain the RAPD score 2 is displayed for one pair of pupil responses to stimulus pattern 2 in cases **a**–**c**, respectively, in Figure 2. RE, right eye 2-s stimulus; LE, left eye 2-s stimulus; Colored A, a straight line connecting the relative pupil diameter at the start of pupil diameter change due to right eye stimulus and that due to the next left eye stimulus; Colored B, a straight line connecting the relative pupil diameter at the start of pupil diameter change due to left eye stimulus and that due to the next right eye stimulus. Red line with red A and B, pupil change in right eye; blue line with blue A and B, pupil change in left eye. If the slope of red line A is a, the slope of blue line A is a′, the slope of red line B is b, and the slope of blue line B is b′; RAPD score 2 is calculated as 10 × log_10_ 10^(b + b′)/2^/10^(a + a′)/2^.

Here, od is the average rate of change in the relative pupil diameter for each eye (the pre-stimulus pupil diameter minus the minimum pupil diameter, which was divided by the pre-stimulus pupil diameter and then multiplied by 100 to be expressed as a percentage) during right eye stimulation, and os is the rate of change in the relative pupil diameter for each eye during left eye stimulation (Figure 3a–c). Positive values indicate relative abnormalities in the left afferent pathway, and negative values indicate relative abnormalities in the right.

RAPD for the second stimulus pattern was calculated based on the binocular average of the relative pupil diameters at the onset of constriction or mydriasis to the right eye stimulus and that to the left eye stimulus. The software automatically determines the rapid change in pupil diameter that occurs 0.15–0.25 s after light stimulation and whether the pupil diameter tends to become smaller (constricted) or larger (mydriatic). The rate of change over time of the pupil diameter relative to that without stimulation before the start of the first stimulation pattern is graphed, and a straight line is drawn connecting the relative pupil diameter of each eye at the start of the pupil diameter change to the right eye stimulation, and the relative pupil diameter of each eye at the start of the pupil diameter change to the left eye stimulation (Figure 3d–f). This curve theoretically has a slope of zero if there is no difference in the amount of constriction and degree of maintenance of constriction between the direct and indirect reflexes of the two eyes (Figure 3f), whereas, if there is a difference, it becomes a negative slope when recording the direct reflex of the eye with better function (indirect reflex of the eye with poorer function) and a positive slope when recording the direct reflex of the eye with poorer function (indirect reflex of the eye with poorer function) (Figure 3e,f). Based on the slope of this drawing line, RAPD score 2 was calculated using the following formula:

RAPD score 2 = 10 × log_10_ 10^(b + b′)/2^/10^(a + a′)/2^

Here, a is the slope of the right eye’s drawn line during right eye stimulation, a′ is the slope of the left eye’s drawn line during right eye stimulation, b is the slope of the left eye’s drawn line during left eye stimulation, and b′ is the slope of the right eye’s drawn line during left eye stimulation. As in the case with RAPD score 1, positive values indicate relative abnormalities in the left afferent pathway, and negative values indicate relative abnormalities in the right afferent pathway.

### 2.4. OCT and OCTA Measurement

**Figure 4:**
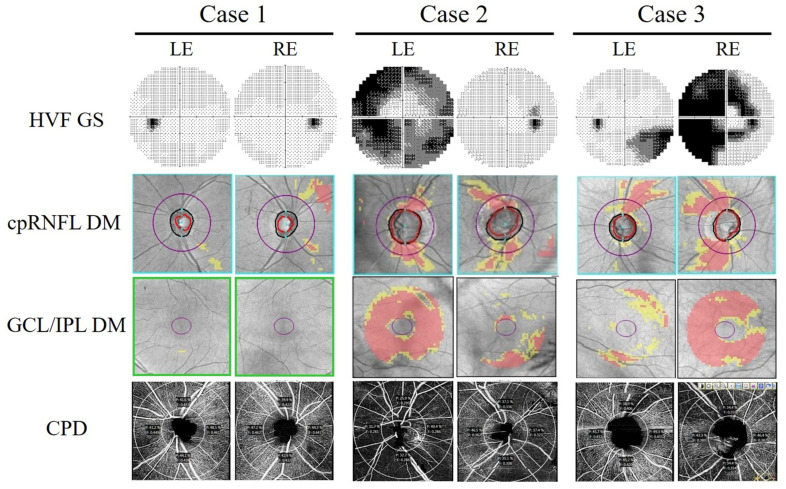
Visual field, optical coherence tomography, and optical coherence tomography angiography findings of representative cases. Grayscale (GS) images of the Humphrey visual field (HVF) test, circumpapillary retinal nerve fiber layer deviation map (cpRNFL DM), ganglion cell layer/inner plexiform layer deviation map (GCL/IPL DM), and radial peripapillary capillary perfusion density (CPD) in cases 1–3, corresponding to a–c, respectively, in Figure 2 and Supplementary Materials Videos S1–S3, respectively. Purple circles correspond to scan areas with a diameter of 3.46 mm for cpRNFLT analyses.

cpRNFLT and macular GCL/IPLT were evaluated under mydriasis using a Cirrus HD-OCT (software version 6.1.0.96; Carl Zeiss Meditec) [23]. The optic nerve head (ONH) protocol employed in the Cirrus HD-OCT system scans a 6 × 6-mm^2^ area centered on the optic disk in three dimensions. cpRNFLT was evaluated by automatically scanning a circular area 3.46 mm in diameter around the optic disk (Figure 4). Images with a signal intensity < 6 were excluded. The average cpRNFLT values were used for the analysis. Ganglion cell analysis was used to analyze the macular inner retinal thickness, which detects and measures GCL/IPLT within a 14.13-mm^2^ ellipsoid region centered on the central fovea (Figure 4). The average GCL/IPLT value was used for the analysis.

Each patient underwent an OCTA scan of a 4.5 × 4.5-mm area centered on the center of the optic disk. Imaging was performed by an experienced technician using a Cirrus HD-OCT 5000 with AngioPlex (Carl Zeiss Meditec, Version 11.0.0.29946) [24]. Image sets were manually reviewed by the researchers for images with insufficient signal strength (<7/10) and segmentation, projection, or motion artifacts. The image quality examiner (M.S.) was blinded to the results of the electronic pupillometer recording. In this study, peripapillary capillary perfusion density (CPD) was measured in the radial peripapillary capillary plexus, which was isolated as a tissue slab containing the retinal layer between the inner limiting membrane and the outer border of the retinal nerve fiber layer (Figure 4). The AngioPlex software converted the en face image into a binary map wherein each pixel was assigned to either a vessel or a non-vessel. The resulting binary map was further skeletonized into a vascular network of 1 pixel width. To exclusively quantify perfusion at the capillary level, vessels with a width > 32 μm before skeletonization were excluded. CPD values were extracted by software from the binary map and vascular skeleton within an area limited to the peripapillary region, which was defined as a ring-shaped region of interest surrounded by a circle with an outer diameter of 4.5 mm and an inner diameter of 2.0 mm centered on the optic disk. Measurements were automatically reported as the average of four (upper, nasal, temporal, and lower) sectors; CPD was calculated as the percentage of pixels in the binary map that were included in the vascular skeleton.

### 2.5. Statistical Analysis

Asymmetry of cpRNFLT, GCL/IPLT, and CPD between the left and right eyes was defined as the percentage difference between the two eyes divided by the value in the eye with the higher value. Asymmetry of visual acuity and visual field loss between the left and right eyes was defined as the difference in logMAR, MD, and VFI. The Shapiro–Wilk test was used for the normality test. Age was expressed as the mean and standard deviation (SD). Variables other than age had either positive or negative values depending on which eye was more severely affected; their absolute values were expressed as the median and interquartile range (IQR). Comparisons of continuous variables between groups with and without RAPD determined by the SFLT were performed with Student’s *t*-test for age and the Mann–Whitney test for all other variables. Comparisons of categorical variables were performed with the Chi square test. The correlation between RAPD scores 1 and 2 was assessed by Pearson correlation coefficient analysis. Receiver operating characteristic (ROC) curve analysis was performed to determine the ability of RAPD scores 1 and 2 to discriminate between the presence and absence of RAPD as determined by SFLT, and the area under the ROC curve (AUC) was calculated. The relationship between asymmetry of functional or structural change and RAPD score 1 or score 2 was examined by linear regression analysis. Statistical analyses were performed using the MedCalc software version 20.210 (MedCalc Software, Ostend, Belgium). *p* < 0.05 was considered significant.

## 3. Results

### 3.1. Patient Demographics

Of the 81 patients, 39 had no RAPD and 42 had RAPD on SFLT (Table 1). The RAPD-negative group consisted of 13 men and 26 women, while the RAPD-positive group consisted of 23 men and 19 women; there was no difference in the proportion of men and women between the two groups (Chi square test; *p* = 0.054). Additionally, there were no significant differences between the two groups in terms of age or absolute values of left–right differences in logMAR. Meanwhile, the absolute values of MD, VFI, cpRNFLT and GCL/IPLT, CPD asymmetry, and RAPD scores 1 and 2 were all significantly greater in the RAPD-positive group than in the RAPD-negative group.

### 3.2. Association between RAPD Scores 1 and 2 and Their Discriminatory Ability for SFLT RAPD

**Figure 5:**
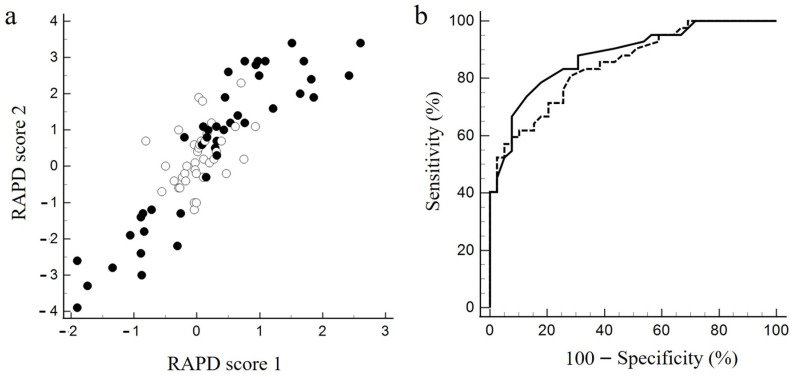
Correlation of two Hitomiru RAPD scores and their discriminative ability against RAPD detected by SFLT. (**a**) Scatterplot of RAPD score 2 against RAPD score 1. White circles, RAPD-negative cases on SFLT. Black circles, positive cases. Positive values for both scores indicate relative abnormalities in the left afferent pathway; negative values indicate relative abnormalities in the right afferent pathway. (**b**) Receiver operating characteristic curves based on SFLT results for RAPD scores 1 and 2. Dotted line, RAPD score 1; solid line, RAPD score 2.

RAPD scores 1 and 2 showed a high correlation coefficient of 0.86 (95% confidence interval [CI], 0.79–0.91, *p* < 0.0001) (Figure 5a). The AUC for RAPD score 1 was 0.85 (95% CI, 0.75–0.92), and that for RAPD score 2 was 0.88 (95% CI, 0.79–0.94), indicating almost equally high detection power (Figure 5b). For RAPD score 1, at a cutoff value of 0.29, the sensitivity, specificity, and Youden index were 81.0%, 71.8%, and 0.53, respectively; at a cutoff value of 1.1, these values were 73.8%, 87.2%, and 0.61%, respectively, for RAPD score 2.

### 3.3. Relationships of RAPD Scores 1 and 2 and Functional and Structural Asymmetry

**Figure 6:**
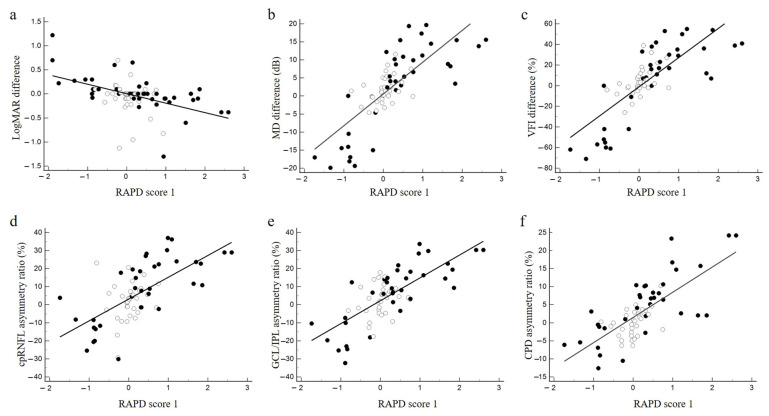
Correlation between functional and structural asymmetry and RAPD score 1. (**a**) Correlation between the difference in logMAR visual acuity of the left and right eyes and RAPD score 1. (**b**) Correlation between the difference in mean deviation (MD) of the left and right eyes and RAPD score 1. (**c**) Correlation between the difference in visual field index (VFI) of the left and right eyes and RAPD score 1. (**d**) Correlation between percentage of right–left asymmetry in mean peripapillary retinal nerve fiber layer (cpRNFL) thickness and RAPD score 1. (**e**) Correlation between percentage of right–left asymmetry in mean GCL/IPL thickness and RAPD score 1. (**f**) Correlation between percentage of right–left asymmetry in mean peripapillary capillary density (CPD) and RAPD score 1. White circles, RAPD-negative cases in the SFLT. Black circles, RAPD-positive cases. Straight lines indicate linear regression lines.

**Figure 7:**
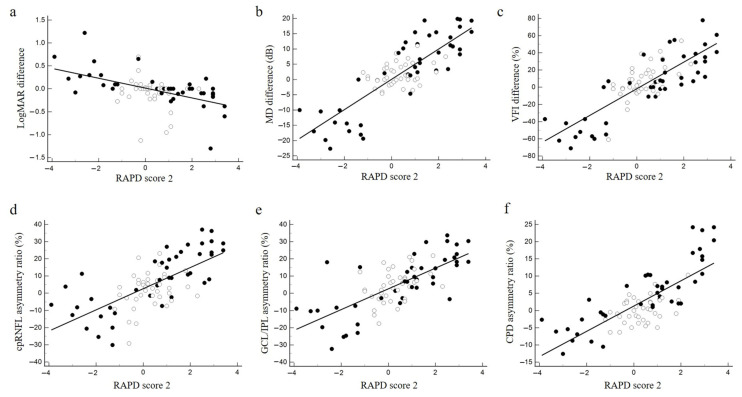
Correlation between functional and structural asymmetry and RAPD score 2. (**a**) Correlation between the difference in logMAR visual acuity of the left and right eyes and RAPD score 2. (**b**) Correlation between the difference in mean deviation (MD) of the left and right eyes and RAPD score 2. (**c**) Correlation between the difference in visual field index (VFI) of the left and right eyes and RAPD score 2. (**d**) Correlation between the percentage of right–left asymmetry in mean peripapillary retinal nerve fiber layer (cpRNFL) thickness and RAPD score 2. (**e**) Correlation between the percentage of right–left asymmetry in mean GCL/IPL thickness and RAPD score 2. (**f**) Correlation between the percentage of right–left asymmetry in mean peripapillary capillary density (CPD) and RAPD score 2. White circles, RAPD-negative cases in the SFLT. Black circles, RAPD-positive cases. Straight lines indicate linear regression lines.

The relationship between RAPD scores 1 and 2 and the asymmetry of structure and function is plotted in Figure 6 and Figure 7, respectively, representing a linear regression line. Significance in the coefficient of determination (R^2^) and slope of the regression line between each parameter is listed in Table 2. RAPD scores 1 and 2 showed similar R^2^ values for each functional or structural parameter of asymmetry. Both scores also showed significantly higher R^2^ values for visual field asymmetry than for visual acuity asymmetry among the functional variables. Meanwhile, regarding structural variables, the R^2^ for both scores increased in the order of cpRNFLT < GCP/IPLT < CPD asymmetry, with only CPD asymmetry exceeding 0.5.

## 4. Discussion

In the current study, RAPD scores 1 and 2 were highly correlated with each other. Both scores also showed similarly high discriminatory power for the presence or absence of RAPD as determined by SFLT. Furthermore, both scores correlated best with visual field asymmetry and worst with visual acuity asymmetry. As for the correlation between RAPD and asymmetric structural change, it was higher for GCL/IPLT than cpRNFLT, and even higher for CPD.

Although some argue that electronic pupillometry is more sensitive than SFLT in detecting RAPD [11,22], others argue the opposite [25]. When detecting RAPD, the rapidity and maintenance of constriction of the light-stimulated eye are determined by considering changes over time [2,22]. Meanwhile, the parameters evaluated when detecting RAPD with an electronic pupillometer, such as initial and maximum constriction, re-mydriasis, and the final pupil diameter after re-mydriasis, are cross-sectional in time [9,10,26]. Hence, a diagnostic algorithm that comprehensively incorporates all parameters has not yet been established. The RAPD score in RAPDx^®^ is a parameter that extracts only the maximum pupil constriction (the ratio of the minimum pupil diameter at maximal constriction to the baseline pupil diameter) or latency to the maximum constriction [11,12,13,14], which is mimicked by RAPD score 1 in this study. In contrast, RAPD score 2 is a parameter that focuses on the degree of change in pupil diameter over time caused by alternating the stimulated eye and is intended to mimic clinical SFLT. A recent meta-analysis of 11 studies that included 7271 subjects reported that RAPD detection can discriminate left–right differences in visual dysfunction in patients with glaucoma with sensitivity of 63% and specificity of 93% [5]. Excluding studies evaluating SFLT, the sensitivity and specificity were reported to be 74% and 85%, respectively [5]. In this study, both RAPD scores showed sensitivity and specificity comparable to that in previous reports. However, RAPD score 2 may not ideally capture time-dependent changes in direct and indirect light reflexes. Biological variables may limit the accurate and predictive identification of patients with slight asymmetric optic nerve disease by the true threshold of these clinical tests [15]. Refinement of recordings and a more comprehensive analysis of pupil changes over time by using artificial intelligence may improve the accuracy of RAPD detection by pupillometry.

The existing electronic pupillometers that can detect RAPD are stationary and not portable. Meanwhile, Hitomiru is portable, so it may be useful in quantitatively and objectively detecting RAPD in patients with glaucoma and optic nerve disease, given the convenience of being able to perform assessments not only in the sitting position but also in the supine position [15,16,17]. Furthermore, RAPD score 2 tended to correlate better with asymmetry in functional and structural deficits than RAPD score 1; hence, it is possible that examining the maintenance of constriction over time is more suitable for the detection of RAPD than the maximum amount of constriction for short-term stimuli.

The degree of RAPD correlates more with visual field asymmetry than with visual acuity asymmetry [6,26]. Thus, it seems reasonable that visual field parameters, which reflect the function of a wider retinal area, correlate more strongly with the degree of RAPD than visual acuity, which reflects only the function of the central fovea. Regarding the correlation between the MD difference and RAPD, previous reports showed that the R^2^ ranges from 0.45 to 0.71 [6,12,14,27]; the present results are comparable to these values (Table 2).

Regarding the R^2^ with asymmetry of cpRNFLT and RAPD, the present results are 0.35 for RAPD score 1 and 0.45 for RAPD score 2; previously reported values were 0.35 [14], 0.362 [13], and 0.557 [6]. Except for our previous report [6], wherein the magnitude of RAPD was quantified by SFLT and ND filters, the correlation between the RAPD score in RAPDx^®^ and cpRNFLT asymmetry is approximately R^2^ = 0.35 in all reports. Meanwhile, the R^2^ of GCL/IPLT asymmetry was 0.447 and 0.489 for the current RAPD scores 1 and 2, respectively, which are both higher than the previously reported value of 0.285 [14]. The R^2^ values for the weighted retinal ganglion cell count and combined structure–function index, which are parameters that integrate visual field data and cpRNFLT, and the RAPD score in RAPDx^®^ were 0.492 and 0.484, respectively [13]. Although simple comparisons cannot be made due to differences in enrollment criteria and pupillometer devices across studies, the correlations between OCT parameter asymmetry and the severity of RAPD are generally worse than those between visual field asymmetry and RAPD. Furthermore, RAPD score 2 showed a higher R^2^ for all parameters than RAPD score 1. Regardless, whether RAPD score 2 is a parameter that correlates more closely with structural and functional asymmetry than RAPD score 1 needs to be tested with a larger number of cases.

Another notable result in this study was that the R^2^ between CVD asymmetry and the RAPD score was 0.53 for score 1 and 0.59 for score 2, showing a stronger correlation than that with the OCT parameters. Using the Split Spectrum Amplitude Decorrelation algorithm (SSADA), Liu et al. [28] were the first to report that the peripapillary vessel density in glaucomatous eyes is reduced compared to normal eyes. Wang et al. [29] reported that the papillary flow index and vessel density were reduced in glaucomatous eyes, and that changes in flow index and vessel density correlated with MD, cpRNFLT, and GCL/IPLT. In a study by Chen et al. using the OCT-Micro Angiography Complex algorithm (OMAG) [30], glaucomatous eyes had significantly lower ONH perfusion compared to normal eyes, and a significant correlation was detected between ONH perfusion, disease severity, and structural changes. The Angioplex OCTA used in this study utilizes OMAG, and Van Melkebeke et al. [31] suggested that OMAG may be more sensitive to microvascular density loss than SSADA. Furthermore, OCTA is not only sensitive to retinal lesions in the early stages of the disease but can also detect further damage in extremely atrophic retinas because of its small floor effect [31,32]. Reportedly, vascular density loss is more rapid [33] than GCL thinning in early-stage glaucoma. In other words, before the retinal structure becomes thin, blood flow may already be compromised, and the vascular density detected by OCTA may decrease due to a decrease in retinal metabolism associated with reduced neural activity. Therefore, the OCTA parameters may be an indicator of both structural and functional changes, and, as a result, their asymmetry may be correlated more strongly with the amount of RAPD than the OCT parameters.

This study had several limitations. First, we did not examine the reproducibility of RAPD in this study. In a study by Zheng et al. [34] using the RAPDx^®^ in healthy patients and those with optic neuritis, the interclass coefficient of the RAPD score was moderate, ranging from 0.65 to 0.67. Kawasaki et al. [35] reported that RAPD variability was not minimized until the number of stimulus pairs was increased to 200 or more. However, as Volpe et al. [15] pointed out, it is not realistic to perform as many as 200 or more cycles of photostimulation, resulting in a 15-min time commitment, in daily practice. Reportedly, the combination of 2.8 s of stimulation and 0.2 s of no stimulation, similar to stimulation pattern 2, results in a smaller 95% CI for a smaller number of times than the combination of 0.2 s of stimulation and 2.8 s of no stimulation, similar to stimulation pattern 1 [35]. Thus, the higher specificity of stimulus pattern 2 may support the findings of Kawasaki et al. [35], i.e., that prolonged stimulation, which mimics clinical SFLT, is more reproducible. Further investigations are needed to determine which stimulation pattern is more consistent with daily clinical practice and is a more reproducible quantification of RAPD.

A second limitation is that Hitomiru is a non-projective simultaneous binocular recording device, so it was not possible for the subject to fixate at a distant target during the pupillary response recording. Therefore, the possibility of contamination with near-sighted reactions cannot be ruled out. However, it would also have been difficult to induce an unconscious near-sighted reflex because the camera was placed in a dark room with no target, and the device was placed as near as 3 cm away from the eye. Furthermore, RAPD scores 1 and 2 showed a strong correlation, suggesting that the influence of the near-vision reflex, if present, could be small.

A third limitation is that normal subjects were not included, and the ability of the electronic pupillometer to detect RAPD was evaluated based on the presence of RAPD by SFLT. In a previous meta-analysis, studies that did not include normal controls were excluded [5]. Not including normal controls may underestimate the ability of electronic pupillometers to detect RAPD. However, it is not known whether electronic pupillometry is more accurate than clinical SFLT; it has been pointed out that the former may have a higher false positive rate [22].

## 5. Conclusions

Hitomiru, which is a portable binocular simultaneous recording electronic pupillometer, showed high RAPD detection capacity when based on clinical RAPD as detected by SFLT. The magnitude and direction of the two independent RAPD scores correlated with the asymmetry of visual function and the retinal structure. CPD asymmetry measured using OCTA may correlate better with RAPD than asymmetry in OCT parameters.

## Figures and Tables

**Table 1:** Summary of patients’ demographics.

	RAPD-Negative Group (*n* = 39)				RAPD-Positive Group (*n* = 42)				
	Mean	SD	Median	IQR	Mean	SD	Median	IQR	*p*-Value
Age	57.8	14.5			60.8	13.9			0.34 *
Abs logMAR Diff.			0.097	0.000–0.176			0.097	0.000–0.273	0.168
Abs MD Diff. (dB)			2.46	0.65–4.49			11.41	6.65–16.95	<0.0001
Abs VFI Diff. (%)			7.00	2.00–14.50			37.00	17.00–54.00	<0.0001
Abs cpRNFLT Ratio (%)			5.71	2.82–9.92			14.20	8.07–24.00	<0.0001
Abs GCL/IPLT Ratio (%)			5.71	3.23–9.97			14.75	8.33–21.84	<0.0001
Abs CVD Ratio (%)			2.63	1.52–3.76			6.92	2.67–10.55	<0.0001
Abs RAPD Score 1			0.18	0.04–0.35			0.85	0.32–1.34	<0.0001
Abs RAPD Score 2			0.60	0.20–0.98			1.90	1.10–2.80	<0.0001

RAPD, relative afferent pupillary defect; SD, standard deviation; IQR, interquartile range; Abs, absolute value; LogMAR, logarithm of minimal angle resolution; MD, mean deviation; VFI, visual field index; cpRNFLT, circumpapillary retinal nerve fiber layer thickness; GCL/IPLT, ganglion cell layer/inner plexiform layer thickness; CVD, capillary vessel density; Diff., difference between right and left eyes; Ratio, ratio of difference between right and left eyes divided by better eye. *, Student’s *t*-test. Comparisons of other variables were made by Mann–Whitney U-test.

**Table 2:** Coefficient of determination of functional and structural asymmetrical variables with RAPD scores 1 and 2.

Independent Variables	Dependent Variables	
	RAPD Score 1	RAPD Score 2
logMAR difference	0.22	0.24
MD difference	0.63	0.67
VFI difference	0.62	0.65
cpRNFLT ratio	0.35	0.45
GCL/IPLT ratio	0.45	0.49
CVD ratio	0.53	0.59

RAPD, relative afferent pupillary defect; LogMAR, logarithm of minimal angle resolution; MD, mean deviation; VFI, visual field index; cpRNFLT, circumpapillary retinal nerve fiber layer thickness; GCL/IPLT, ganglion cell layer/inner plexiform layer thickness; CVD, capillary vessel density. All had *p* < 0.0001.

## Data Availability

All data generated or analyzed during this study are included in this article. Further inquiries can be directed to the corresponding author.

## Supplementary Materials

The following supporting information can be downloaded at: https://www.mdpi.com/article/10.3390/jcm12123936/s1, Video S1: A representative case (Case 1) with no RAPD according to Hitomiru. Video S2: A representative case (Case 2) with RAPD in the left eye according to Hitomiru. Video S3: A representative case (Case 3) with RAPD in the right eye according to Hitomiru.
